# Suspected Acute Respiratory Distress Syndrome Associated With the Use of Intravenous Lipid Emulsion Therapy in a Dog: A Case Report

**DOI:** 10.3389/fvets.2019.00225

**Published:** 2019-07-09

**Authors:** Heike Botha, Samuel Hollis Jennings, Saya A. Press, Stephanie A. Istvan

**Affiliations:** ^1^ECC Resident at The Veterinary Specialty Hospital, San Diego, CA, United States; ^2^MSpVM, DACVP Diagnostic Pathologist, Ethos Diagnostic Science, San Diego, CA, United States; ^3^DACVECC at The Veterinary Specialty Hospital, San Diego, CA, United States

**Keywords:** intravenous lipid emulsion toxicity, toxicosis, tetrahydrocannabinol, acute respiratory distress syndrome, ARDS, adverse drug reaction, canine

## Abstract

A 12-year-old male neutered Bichon Frise presented to the Emergency Department for stupor and bradycardia after ingestion of chocolate covered 450 mg (90 mg/kg) tetrahydrocannabinol. The patient was hospitalized for supportive care, IV fluid therapy and monitoring in the intensive care unit. During hospitalization the patient became comatose and bradypneic. Treatment with intravenous lipid emulsion (ILE) therapy was instituted to accelerate toxin elimination, reduce the risk of complications related to progressive obtundation and shorten hospitalization time. Five hours after infusion, the patient developed severe respiratory distress and was ultimately euthanized. Post-mortem histologic evaluation of lung revealed severe pulmonary edema consistent with acute respiratory distress syndrome. There are infrequent reports of adverse effects associated with ILE therapy for toxicosis in veterinary medicine despite reports of complications such as acute respiratory distress syndrome in human literature. The purpose of this report is to describe the potential for a severe adverse event after treatment of a toxicosis with ILE therapy.

## Background

Intravenous lipid emulsion (ILE) has historically been used in parenteral nutritional as a source of fatty acids in hospitalized patients. ILE is gaining interest as an antidote in lipophilic drug intoxications (1, 2). After its establishment as an effective treatment in murine and canine models, ILE was first used in treatment of acute local anesthetic systemic toxicities (LAST) in people (3, 4). Subsequently, numerous case reports were published describing the successful use of ILE therapy in neurologic, cardiac and non-steroidal anti-inflammatory drug toxicities in veterinary medicine (4–11).

Reported adverse events associated with ILE infusions are sporadic or extrapolated from its use in parenteral nutrition, there is a case report of suspected acute respiratory distress syndrome (ARDS) secondary to the use of ILE for verapamil toxicosis (12). The adverse events reported with ILE infusions include phlebitis, immunosuppression, cardiovascular, lipid-emboli, hemolysis, acute kidney injury, metabolic acidosis, pulmonary complications (acute lung injury, ARDS, hypoxia and ventilation-perfusion mismatch), pancreatitis or fat overload syndrome, hypersensitivity or allergic reactions and vomiting, persistent gross lipemia and suspected corneal lipidosis (2, 7, 9–11, 13–19). There is a paucity of reports of adverse events or mortality related to ILE use in veterinary medicine. This case describes the suspected occurrence of ARDS secondary to ILE administration in a dog.

## Case Presentation

A 5.16 kg, 12-year-old male neutered Bichon Frise was evaluated approximately 14 h after ingestion of a dark chocolate bar containing 450 mg tetrahydrocannabinol (THC, 90 mg/kg). The presenting complaint was profound sedation. No vomiting or regurgitation was noted at home.

At presentation to the Emergency Department, the patient was stuporous with a reduced to absent gag reflex, and globe position was ventromedial bilaterally (OU). Rectal temperature was 99.0°F (37.2°C). Cardiorespiratory auscultation was unremarkable; however, his respiratory rate was 12 breaths/min with shallow chest excursions and his heart rate was 56 beats/min. Initial venous blood gas demonstrated a respiratory acidosis with only a mild increase in plasma bicarbonate concentration suggesting an acute process (Table 1). Doppler blood pressure was 160–170 mmHg. The patient was hospitalized in intensive care unit (ICU) for monitoring, seizure watch and fluid therapy (36 mL/h for 8 h and then 18 mL/h for 5 h) after receiving a single injection of 1 mg/kg maropitant intravenously (Cerenia; Zoetis, Parsippany, NJ, USA) to decrease the risk of vomiting and consequent aspiration pneumonia.

**Table 1:** iStat obtained at presentation, venous blood sample (Abbott Heska i-STAT Veterinary Clinical Analyzer, Abaxis North America, Union City, CA, USA).

pH	7.198
pCO_2_	68.5 mmHg
pO_2_	48 mmHg
HCO^3−^	26.6 mmol/L
Lac	0.86 mmol/L
AG	2 mmol/L
HCT	49 %
Na	138 mmol/L
K	5.1 mmol/L
Cl	119 mmol/L
iCa	0.9 mg/dL
Glucose	102 mg/dL
BUN	11 mg/dL
Crea	0.6 mg/dL
BE	−1 mmol/L
PCV	55%
TP	7.1 g/dL

During the first 8 h of hospitalization, the patient's neurologic status progressed from stuporous to comatose. The respiratory pattern became shallower (24 breaths/min), while the rectal temperature decreased to 96.8°F (36°C). Due to financial limitations and inability to hospitalize the patient in ICU for an extended time, ILE therapy was initiated in an attempt to shorten hospitalization time (2, 3, 20).

Baseline vital parameters (Table 2) were obtained and then an ILE protocol derived from human dosing recommendations was initiated. An initial 20% sterile lipid (INTRALIPID 20% IV Fat Emulsion; 500 mL, Baxter Healthcare, Deerfield, IL, USA) bolus of 7.5 mL (1.4 mL/kg) over 10 min was administered through an in-line 1.2 micron filter intravenously. This was followed by an intravenous constant rate infusion (CRI) of 0.16 mL/kg/min over 1 h. Serum was assessed at 30 and 60 min after discontinuation of ILE by centrifugation of a heparinized micro-hematocrit capillary tube (Heparinized micro-hematocrit capillary tube, Kimble-Chase, Rockwood, TN, USA) with visual assessment of lipemia. Serum was evaluated to be lipemic at both time points. Intravenous fluid therapy was resumed after a total ILE infusion volume of 57 mL.

**Table 2:** Vital parameter trends noted on the Infusion Monitoring Sheet.

**Parameter**	**Before ILE, at the start of infusion**	**3 h after initiation of ILE**
Temperature (°C)	37.1	39.2
Pulse (beats/min)	80	112
Respiratory rate (breaths/min)	24	42
Blood pressure (mmHg, Doppler)	200	220

The patient became alert and responsive to stimuli during the ILE CRI. Heart rate and temperature increased compared to baseline [80 beats/min, 98.9°F (37.1°C), respectively]. The patient developed frequent episodes of liquid diarrhea 1 h after finishing the ILE CRI. At 5 h post-ILE infusion, the patient became acutely tachypneic and developed progressive respiratory distress. Respiratory rate was 140 breaths/min with increasing effort that progressed to orthopnea with gray mucous membranes. Auscultation revealed loud, diffuse crackles bilaterally. Flow-by supplemental oxygen was provided and a pulse oximeter reading obtained with oxygen therapy was 90%. Serum remained markedly lipemic at this time. The patient developed a severe productive cough, producing increasing amounts of a white foamy fluid at which point he was endotracheally intubated. Bedside ultrasound revealed scant pleural effusion and no left atrial enlargement assessed using left atrial to aorta ratio (stated as “not enlarged” in the medical record, considered to be an left atrial to aorta ratio <1.5). Doppler blood pressure was 50 mmHg. At this point the decision to euthanize was made due to financial limitations in the face of progressive decline. A large amount of pink tinged foamy white fluid was dumped from the endotracheal tube after euthanasia. Pulmonary fluid was evaluated and total protein (TP) obtained via refractometer was 5.4 g/dL (resulting in an edema fluid-to-plasma protein ratio of 0.76). A sterile sample of the 20% IV lipid emulsion used in this patient was negative for aerobic and anaerobic growth at 72 h.

Postmortem exam revealed a scant amount of clear pleural effusion and foamy fluid throughout the lower airways. The lungs were heterogeneously wet and heavy, supporting pulmonary edema. There was no gross evidence of hemorrhage or aspirated stomach contents.

**Figure 1:**
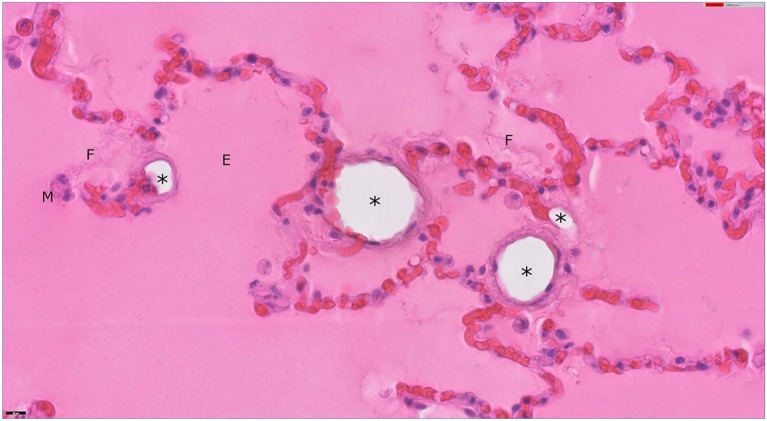
Lung from a dog. Alveoli are filled with eosinophilic, proteinaceous, edema fluid (E) with strands of fibrin (F). A few, foamy alveolar macrophages (M) are present. Several alveolar septa in this field are distended by clear, round, empty spaces (^*^) that appear to be intravascular and could present extracted lipid emboli. Hematoxylin and eosin stain; bar = 20 μm.

Six sections from affected areas of lung were examined histologically by a board-certified veterinary anatomic pathologist following routine processing and hematoxylin and eosin staining (Figure 1). A large proportion of the alveoli contained proteinaceous fluid admixed with small to moderate amounts of fibrin, moderate numbers of foamy macrophages, and occasionally small number of neutrophils, consistent with diffuse alveolar damage. Occasionally, alveolar septa were distended by discrete, round to tubular, empty spaces that appear to be intravascular and could represent lipid emboli. However, at the time of manuscript preparation, unprocessed lung sample were no longer available to pursue special stains for lipid on frozen sections. The finding of diffuse alveolar damage correlates with the clinical diagnosis of ARDS. The potential lipid emboli may have been additional drivers or contributors to impaired pulmonary physiology.

## Discussion

This case describes an adverse event following chocolate ingestion and THC intoxication and the use of ILE therapy. After considering potential causes for the clinical deterioration, the authors suspect that development of ARDS secondary to ILE infusion resulted in acute decline of the patient. Diagnosis was based on the exclusion of other risk factors that have the potential of inducing acute lung injury.

Tetrahydrocannabinol is highly lipophilic (partition coefficient (*P*) value >6,000) and is distributed in adipose tissue, liver, lungs and spleen after absorption from the gastrointestinal tract. THC is slowly redistributed into plasma before excretion in feces and urine (21, 22). The lipophilicity provides a theoretical justification for use of ILE in treatment of THC toxicosis. In this case, initial improvement was noted in mentation when the patient became more responsive, but the patient subsequently developed severe respiratory distress. Criteria implicating ARDS as the cause of respiratory distress in this patient include the acute onset of clinical signs, known risk factor (ILE infusion), proteinaceous fluid in conducting airways and neutrophilic inflammation in pulmonary parenchyma. Although not traditionally considered a component of the definition of VetALI/VetARDS the finding of an edema fluid-to-plasma protein ratio ≥0.65 (TP edema fluid 5.4 g/dL and plasma protein 7.1 g/dL, ratio 0.76) makes cardiogenic pulmonary edema less likely and may indicate increased alveolar capillary membrane permeability (23, 24).

ILE infusions are used as an antidote for non-local anesthetic agents (1, 2, 20, 25). Though theoretical, there are two proposed mechanisms of ILE's antidotal properties. In the lipid sink theory the offending agent is partitioned into the intravascular lipid phase, resulting in compartmentalization of highly soluble agents, removing lipid soluble toxins from their sites of action and allowing metabolism and excretion (2, 3, 20). Furthermore, this partition results in a concentration gradient exerting a pull of the agent from tissues into the vascular compartment, accelerating removal from the interstitial space (20). An alternative theory addresses previously observed improved cardiac performance after infusion of ILE and suggests that an increase in fatty acids provides an energy source for cardiac myocytes, improving cardiac myocyte survival and counteracting cardio-toxic effects of LAST that may include inhibition of fatty acid transport into mitochondria (2, 3, 17, 20). Thus, ILE infusions may provide a benefit in toxicosis with lipophilic agents and in agents that impair mitochondrial metabolism (25).

Reports of ILE treatment in non-LAST in veterinary medicine have documented favorable outcomes in treatment of calcium channel blocker, bromethalin, avermectin parasiticides, baclofen, bupropion, loperamide, permethrin (cats), and sertraline toxicities (6, 11, 13–17, 20, 21, 26). The reported adverse events in these cases are limited to extravasation and increased toxin levels (11, 14). Case reports are available for use of ILE therapy for non-LAST in people in toxicosis resulting from tricyclic-antidepressant, calcium channel blocker, parasiticides, herbicides, and other psychotropic agents, though controlled studies are lacking (2, 20).

Unfortunately, when ILE is used as a treatment it can be difficult to delineate a complication of therapy vs. a complication from underlying intoxication, which should be considered in the case described here. Deleterious pulmonary effects documented in human medicine and animal studies are predominantly thought to include deposition of lipid in the vasculature and reticuloendothelial system and an increase in fatty acids providing a substrate for pro-inflammatory prostaglandin pathways, which leads to changes in the vascular tone and disruption of alveolar capillary membranes (13, 17, 27). These effects were not noted in clinical studies with baseline healthy lungs but were limited to critically ill or septic animal models (27, 28). There is evidence to suggest that the incidence of ARDS secondary to ILE infusion may be associated with CRI rate and total dose (13). Furthermore, lipid droplets larger than 1 μm in size have the potential to cause obstruction of microcirculation after phagocytosis by the reticuloendothelial system, initiating an inflammatory response (25).

The goal of ILE infusion in toxicosis it to achieve a sustained lipemic plasma phase without inducing fat overload, however, there are established infusion protocols only for LAST in people (29). No standardized or optimal dosing protocol has been studied for treatment of non-LAST (17, 20). Current recommended protocol for LAST, which is commonly used in other toxicities, is an initial bolus of 1.5 mL/kg 20% intralipid solution over 1 min followed by a CRI of 0.25–0.5 mL/kg/min until circulatory stability is achieved (17, 20, 29). Variations of this protocol have been used in veterinary case reports. While doses vary, they all use a 20% intralipid loading bolus followed by a CRI, with monitoring of serum for development of gross lipemia. The lack of clinical studies in veterinary medicine, emphasizes the importance of a careful risk-benefit assessment before instituting therapy and diligent pharmacovigilance in reporting of adverse events.

There are reports implicating ARDS as a potential adverse event associated with ILE infusions in people, however most of the patients were critically ill, making it hard to discern whether ARDS resulted from ILE infusion or was a result of underlying illness (12, 27, 30). The authors do not believe that this patient had an additional condition predisposing to the development of ARDS, though this cannot be entirely ruled out. Respiratory changes noted in prior adverse event reports include an increase in mean pulmonary arterial pressure, increased venous admixture, decreased PaO_2_:FiO_2_ (ratio of arterial oxygen partial pressure to fractional inspired oxygen) and an increased Aa-gradient (Alveolar-arterial oxygen concentration gradient) (27, 30). These changes resolved in one study after discontinuation of ILE therapy (27). Unfortunately, this data was not obtained for the patient in this study, however close temporal relation to ILE infusion makes it highly likely that the observed deterioration was related to this intervention. Post mortem findings were suggestive of ARDS as the cause of respiratory compromise.

Extrapolation from TPN cases suggests that adverse effects could occur with use of ILE in the treatment of toxicities. Potential for significant adverse effects seems to be associated with higher doses and more rapid infusion rates (13). The authors believe there may be a significant publication bias relating to adverse events associated with use of ILE therapy as an antidote. Literature in veterinary medicine is limited to case reports, resulting in a tendency to only report successful outcomes and an absence of reporting of unfavorable outcomes.

The primary limitation of this case report is common to all descriptive studies: causal inference is not possible from uncontrolled observations. Thus, it cannot be excluded that ARDS was secondary to THC or chocolate intoxication, or an adverse drug reaction to other treatments administered (i.e., maropitant). A literature search using the search engines Google Scholar, PubMed and Microsoft Academic was performed using keywords and MeSH terms including chocolate, theobromine, methylxanthines, maropitant, ARDS, respiratory, edema, dog, canine, veterinary, THC, tetrahydrocannabinol, respiratory distress, and pulmonary complications did not reveal any previous reports indicating a similar causality.

Airway complications secondary to THC inhalation (not ingestion) in people have been reported in conjunction with tainted cigarettes or chronic exposure (31, 32). To the authors knowledge, there are no reports of similar respiratory complications in dogs secondary to chocolate intoxication or maropitant use. The pulmonary effects of intravenous theophylline and a methyxanthine derivative (S9795) in dogs were decreased arterial partial pressure of oxygen without change in lung mechanics and decreased compliance, respectively (33). Thus, the potential for methylxanthine induced changes in lung function cannot be completely excluded. It is the authors' opinion that the lack of other clinical signs indicating methylxanthine intoxication makes this possibility less likely.

Although a cardiologist did not evaluate this patient for underlying cardiac disease, history, physical examination and bedside ultrasound were not consistent with underlying structural heart disease nor volume overload. In addition, total fluid rate (352 mL, 5.8 mL/kg/h) of crystalloids or total fluid load including ILE (409 mL, 6.6 mL/kg/h) respectively, was appropriate and not likely to result in fluid overload.

The causality of ILE infusion and development of ARDS cannot be proven in this case, however, temporal association and biological plausibility should bring into question the use of ILE as a treatment in benign intoxications. To the authors' knowledge, this is the first report of a case of suspected ARDS secondary to ILE infusion in veterinary medicine. Prospective, controlled studies are lacking and are needed to more accurately assess risks of this therapy option. Until this time, treatment decisions may need to be reserved for life-threatening intoxications with lipid-soluble agents, after exhaustion of traditional established therapeutic protocols. Client education poses an important aspect in veterinary medicine and should precede the use of ILE therapy as an antidote for toxicosis in all instances. Current ILE administration guidelines are extrapolated from human medicine protocols and are predominantly empiric. Controlled studies in animals may assist in determination of safe and effective dosage recommendations, duration of therapy and threshold for potential complications but are challenging to accomplish. Furthermore, patients treated with ILE should be very closely monitored for development of complications.

## Ethics Statement

The dog detailed in the case report presented as a patient to the Veterinary Specialty Hospital in San Diego, CA. The clients signed a consent form to permit hospitalization and treatment. Additional consent was obtained for anonymized necropsy and post-mortem samples for research purposes.

## Author Contributions

HB and SI were involved in clinical management of the case. All authors were involved in the preparation of the manuscript, contributed to manuscript revision, and approved the submitted version.

### Conflict of Interest Statement

The authors declare that the research was conducted in the absence of any commercial or financial relationships that could be construed as a potential conflict of interest.

## References

[1] Zyoud SEH Waring WS Al-Jabi SW Sweileh WM Rahhal B Awang R. Intravenous lipid emulsion as an antidote for the treatment of acute poisoning: a bibliometric analysis of human and animal studies. Basic Clin Pharmacol Toxicol. (2016) 119:512–9. 10.1111/bcpt.12609 27098056

[2] Gwaltney-Brant S Meadows I. Use of intravenous lipid emulsions for treating certain poisoning cases in small animals. Vet Clin Small Anim Pract. (2012) 42:251–62. 10.1016/j.cvsm.2011.12.001 22381177

[3] Cao D Heard K Foran M Koyfman A. Intravenous lipid emulsion in the emergency department: a systematic review of recent literature. J Emerg Med. (2015) 48:387–97. 10.1016/j.jemermed.2014.10.009 25534900

[4] Weinberg G Ripper R Feinstein DL Hoffman W. Lipid emulsion infusion rescues dogs from bupivacaine-induced cardiac toxicity. Regional Anesthesia Pain Med. (2003) 28:198–202. 10.1097/00115550-200305000-00005 12772136

[5] Clarke DL Lee JA Murphy LA Reineke EL. Use of intravenous lipid emulsion to treat ivermectin toxicosis in a Border Collie. J Am Vet Med Assoc. (2011) 239:1328–33. 10.2460/javma.239.10.1328 22044330

[6] O'Brien TQ Clark-Price SC Evans EE Di Fazio R McMichael MA. Infusion of a lipid emulsion to treat lidocaine intoxication in a cat. J Am Vet Med Assoc. (2010) 237:1455–8. 10.2460/javma.237.12.1455 21155686

[7] Bates N Chatterton J Robbins C Wells K Hughes J Stone M Lipid infusion in the management of poisoning: a report of 6 canine cases. Vet Record Case Rep. (2013) 1:e101036 10.1136/vetreccr.101036rep 23423482

[8] Maton BL Simmonds EE Lee JA Alwood AJ. The use of high-dose insulin therapy and intravenous lipid emulsion to treat severe, refractory diltiazem toxicosis in a dog. J Vet Emerg Crit Care. (2013) 23:321–7. 10.1111/vec.12053 23656275

[9] Bolfer L McMichael M Ngwenyama TR O'Brien MA. Treatment of ibuprofen toxicosis in a dog with IV lipid emulsion. J Am Anim Hosp Assoc. (2014) 50:136–40. 10.5326/JAAHA-MS-5979 24446399

[10] Kuo K Odunayo A. Adjunctive therapy with intravenous lipid emulsion and methocarbamol for permethrin toxicity in 2 cats. J Vet Emerg Crit Care. (2013) 23:436–41. 10.1111/vec.12070 23855545

[11] Brückner M Schwedes CS Successful treatment of permethrin toxicosis in two cats with an intravenous lipid administration. Tierärztliche Praxis Kleintiere. (2012) 2:129–34. 10.1055/s-0038-1623631 22526817

[12] Friedman T Feld Y Adler Z Bolotin G Bentur Y Acute respiratory distress syndrome associated with intravenous lipid emulsion therapy for verapamil toxicity, successfully treated with veno-venous ECMO. J Develop Drugs. (2017) 6:2 10.4172/2329-6631.1000182

[13] Hayes BD Gosselin S Calello DP Nacca N Rollins CJ Abourbih D. Systematic review of clinical adverse events reported after acute intravenous lipid emulsion administration. Clin Toxicol. (2016) 54:365–404. 10.3109/15563650.2016.1151528 27035513

[14] Remold EW Studies of the toxicity of an intravenous fat emulsion. I. Hematologic changes and survival after administration of a soybean oil (FE-S15) in beagles. J Parent Enteral Nutr. (1979) 3:328–34. 10.1177/014860717900300502 117123

[15] Levine M Skolnik AB Ruha AM Bosak A Menke N Pizon AF. Complications following antidotal use of intravenous lipid emulsion therapy. J Med Toxicol. (2014) 10:10–4. 10.1007/s13181-013-0356-1 24338451 PMC3951645

[16] Xenoulis PG Steiner JM. Lipid metabolism and hyperlipidemia in dogs. Vet J. (2010) 183:12–21. 10.1016/j.tvjl.2008.10.011 19167915

[17] Fernandez AL Lee JA Rahilly L Hovda L Brutlag AG Engebretsen K. The use of intravenous lipid emulsion as an antidote in veterinary toxicology. J Vet Emerg Crit Care. (2011) 21:309–20. 10.1111/j.1476-4431.2011.00657.x 21827588

[18] Seitz MA Burkitt-Creedon JM. Persistent gross lipemia and suspected corneal lipidosis following intravenous lipid therapy in a cat with permethrin toxicosis. J Vet Emerg Crit Care. (2016) 26:804–8. 10.1111/vec.12440 26748969

[19] Bass JJr Friedl W Jeranek W. Intralipid causing adult respiratory distress syndrome. J Natl Med Assoc. (1984) 76:401. 6737497 PMC2561677

[20] Rothschild L Bern S Oswald S Weinberg G. Intravenous lipid emulsion in clinical toxicology. Scand J Trauma Resuscit Emerg Med. (2010) 18:51. 10.1186/1757-7241-18-51 20923546 PMC2958894

[21] Huestis MA. Human cannabinoid pharmacokinetics. Chem Biodivers. (2007) 4:1770–804. 10.1002/cbdv.200790152 17712819 PMC2689518

[22] Sharma P Murthy P Bharath MS. Chemistry, metabolism, and toxicology of cannabis: clinical implications. Iranian J Psychiatry. (2012) 7:149. 23408483 PMC3570572

[23] Wilkins PA Otto CM Baumgardner JE Dunkel B Bedenice D Paradis MR Acute lung injury and acute respiratory distress syndromes in veterinary medicine: consensus definitions: the Dorothy Russell Havemeyer Working Group on ALI and ARDS in Veterinary Medicine. J Vet Emerg Crit Care. (2007) 17:333–9. 10.1111/j.1476-4431.2007.00238.x

[24] Ware LB Fremont RD Bastarache JA Calfee CS Matthay MA. Determining the aetiology of pulmonary oedema by the oedema fluid-to-plasma protein ratio. Eur Respir J. (2010) 35:331–7. 10.1183/09031936.00098709 19741024 PMC2819058

[25] Cave G Harvey M. Intravenous lipid emulsion as antidote beyond local anesthetic toxicity: a systematic review. Acad Emerg Med. (2009) 16:815–24. 10.1111/j.1553-2712.2009.00499.x 19845549

[26] Heggem-Perry B McMichael M O'Brien M Moran C. Intravenous lipid emulsion therapy for bromethalin toxicity in a dog. J Am Anim Hosp Assoc. (2016) 52:265–8. 10.5326/JAAHA-MS-6396 27259025

[27] Hwang TL Huang SL Chen MF. Effects of intravenous fat emulsion on respiratory failure. Chest. (1990) 97:934–8. 10.1378/chest.97.4.934 2108849

[28] Turner-Lawrence DE Kerns W. Intravenous fat emulsion: a potential novel antidote. J Med Toxicol. (2008) 4:109–14. 10.1007/BF03160965 18570172 PMC3550130

[29] Gosselin S Hoegberg LC Hoffman RS Graudins A Stork CM Thomas SH. Evidence-based recommendations on the use of intravenous lipid emulsion therapy in poisoning. Clin Toxicol. (2016) 54:899–923. 10.1080/15563650.2016.1214275 27608281

[30] Venus B Smith RA Patel C Sandoval E. Hemodynamic and gas exchange alterations during intralipid infusion in patients with adult respiratory distress syndrome. Chest. (1989) 95:1278–81. 10.1378/chest.95.6.1278 2721266

[31] Gilbert CR Michael B Cavarocchi NC “Smoking wet”: respiratory failure related to smoking tainted marijuana cigarettes. Texas Heart Inst J. (2013) 40:64–7. PMC3568288 23466531

[32] Martinasek MP McGrogan JB Maysonet A. A systematic review of the respiratory effects of inhalational marijuana. Respir Care. (2016) 61:1543–51. 10.4187/respcare.04846 27507173

[33] Lejeune P Naeije R. Effects of progressively increased doses of theophylline and of S 9795 on hemodynamics, blood gases and lung mechanics in dogs. Arch Int Pharmacodyn Ther. (1988) 294:215–27. 3233049

